# Supplementary material for: Individual retrotransposon integrants are differentially controlled by KZFP/KAP1-dependent histone methylation, DNA methylation and TET-mediated hydroxymethylation in naïve embryonic stem cells
Source: Epigenetics Chromatin. 2018 Feb 26;11:7. doi: 10.1186/s13072-018-0177-1 (PMC6389204; doi:10.1186/s13072-018-0177-1)
Supplement: Supplementary file 11 — Additional file 11. Pattern analysis. [file 13072_2018_177_MOESM11_ESM.zip › Patterns analysis/DataTables/extensions/FixedColumns/examples/css_size.html]

FixedColumns example - CSS row sizing


# FixedColumns example CSS row sizing

Because of the method FixedColumns uses to display the fixed columns, an important consideration is
the alignment of the rows due to different heights in the individual rows of the table. There are two
different algorithms in FixedColumns which can be used, or you can switch off automatic row sizing all
together. This is controlled through the `heightMatch` parameter. If can take the following
values:

- `none` - no automatic row height matching is
  performed. CSS can be used in this case and is useful when speed is of primary importance.
- `semiauto` (default) - the height calculation will be
  performed once, and the result cached to be used again (`fnRecalculateHeight` can be
  used to force recalculation)
- `auto` - height matching is performed on every draw
  (slowest but must accurate)

This example shows row height matching switched off but there is a CSS statement of `tr {
height: 50px }` to force all rows to the same height.

| First name | Last name | Position | Office | Age | Start date | Salary | Extn. | E-mail |
| --- | --- | --- | --- | --- | --- | --- | --- | --- |
| Tiger | Nixon | System Architect | Edinburgh | 61 | 2011/04/25 | $320,800 | 5421 | t.nixon@datatables.net |
| Garrett | Winters | Accountant | Tokyo | 63 | 2011/07/25 | $170,750 | 8422 | g.winters@datatables.net |
| Ashton | Cox | Junior Technical Author | San Francisco | 66 | 2009/01/12 | $86,000 | 1562 | a.cox@datatables.net |
| Cedric | Kelly | Senior Javascript Developer | Edinburgh | 22 | 2012/03/29 | $433,060 | 6224 | c.kelly@datatables.net |
| Airi | Satou | Accountant | Tokyo | 33 | 2008/11/28 | $162,700 | 5407 | a.satou@datatables.net |
| Brielle | Williamson | Integration Specialist | New York | 61 | 2012/12/02 | $372,000 | 4804 | b.williamson@datatables.net |
| Herrod | Chandler | Sales Assistant | San Francisco | 59 | 2012/08/06 | $137,500 | 9608 | h.chandler@datatables.net |
| Rhona | Davidson | Integration Specialist | Tokyo | 55 | 2010/10/14 | $327,900 | 6200 | r.davidson@datatables.net |
| Colleen | Hurst | Javascript Developer | San Francisco | 39 | 2009/09/15 | $205,500 | 2360 | c.hurst@datatables.net |
| Sonya | Frost | Software Engineer | Edinburgh | 23 | 2008/12/13 | $103,600 | 1667 | s.frost@datatables.net |
| Jena | Gaines | Office Manager | London | 30 | 2008/12/19 | $90,560 | 3814 | j.gaines@datatables.net |
| Quinn | Flynn | Support Lead | Edinburgh | 22 | 2013/03/03 | $342,000 | 9497 | q.flynn@datatables.net |
| Charde | Marshall | Regional Director | San Francisco | 36 | 2008/10/16 | $470,600 | 6741 | c.marshall@datatables.net |
| Haley | Kennedy | Senior Marketing Designer | London | 43 | 2012/12/18 | $313,500 | 3597 | h.kennedy@datatables.net |
| Tatyana | Fitzpatrick | Regional Director | London | 19 | 2010/03/17 | $385,750 | 1965 | t.fitzpatrick@datatables.net |
| Michael | Silva | Marketing Designer | London | 66 | 2012/11/27 | $198,500 | 1581 | m.silva@datatables.net |
| Paul | Byrd | Chief Financial Officer (CFO) | New York | 64 | 2010/06/09 | $725,000 | 3059 | p.byrd@datatables.net |
| Gloria | Little | Systems Administrator | New York | 59 | 2009/04/10 | $237,500 | 1721 | g.little@datatables.net |
| Bradley | Greer | Software Engineer | London | 41 | 2012/10/13 | $132,000 | 2558 | b.greer@datatables.net |
| Dai | Rios | Personnel Lead | Edinburgh | 35 | 2012/09/26 | $217,500 | 2290 | d.rios@datatables.net |
| Jenette | Caldwell | Development Lead | New York | 30 | 2011/09/03 | $345,000 | 1937 | j.caldwell@datatables.net |
| Yuri | Berry | Chief Marketing Officer (CMO) | New York | 40 | 2009/06/25 | $675,000 | 6154 | y.berry@datatables.net |
| Caesar | Vance | Pre-Sales Support | New York | 21 | 2011/12/12 | $106,450 | 8330 | c.vance@datatables.net |
| Doris | Wilder | Sales Assistant | Sidney | 23 | 2010/09/20 | $85,600 | 3023 | d.wilder@datatables.net |
| Angelica | Ramos | Chief Executive Officer (CEO) | London | 47 | 2009/10/09 | $1,200,000 | 5797 | a.ramos@datatables.net |
| Gavin | Joyce | Developer | Edinburgh | 42 | 2010/12/22 | $92,575 | 8822 | g.joyce@datatables.net |
| Jennifer | Chang | Regional Director | Singapore | 28 | 2010/11/14 | $357,650 | 9239 | j.chang@datatables.net |
| Brenden | Wagner | Software Engineer | San Francisco | 28 | 2011/06/07 | $206,850 | 1314 | b.wagner@datatables.net |
| Fiona | Green | Chief Operating Officer (COO) | San Francisco | 48 | 2010/03/11 | $850,000 | 2947 | f.green@datatables.net |
| Shou | Itou | Regional Marketing | Tokyo | 20 | 2011/08/14 | $163,000 | 8899 | s.itou@datatables.net |
| Michelle | House | Integration Specialist | Sidney | 37 | 2011/06/02 | $95,400 | 2769 | m.house@datatables.net |
| Suki | Burks | Developer | London | 53 | 2009/10/22 | $114,500 | 6832 | s.burks@datatables.net |
| Prescott | Bartlett | Technical Author | London | 27 | 2011/05/07 | $145,000 | 3606 | p.bartlett@datatables.net |
| Gavin | Cortez | Team Leader | San Francisco | 22 | 2008/10/26 | $235,500 | 2860 | g.cortez@datatables.net |
| Martena | Mccray | Post-Sales support | Edinburgh | 46 | 2011/03/09 | $324,050 | 8240 | m.mccray@datatables.net |
| Unity | Butler | Marketing Designer | San Francisco | 47 | 2009/12/09 | $85,675 | 5384 | u.butler@datatables.net |
| Howard | Hatfield | Office Manager | San Francisco | 51 | 2008/12/16 | $164,500 | 7031 | h.hatfield@datatables.net |
| Hope | Fuentes | Secretary | San Francisco | 41 | 2010/02/12 | $109,850 | 6318 | h.fuentes@datatables.net |
| Vivian | Harrell | Financial Controller | San Francisco | 62 | 2009/02/14 | $452,500 | 9422 | v.harrell@datatables.net |
| Timothy | Mooney | Office Manager | London | 37 | 2008/12/11 | $136,200 | 7580 | t.mooney@datatables.net |
| Jackson | Bradshaw | Director | New York | 65 | 2008/09/26 | $645,750 | 1042 | j.bradshaw@datatables.net |
| Olivia | Liang | Support Engineer | Singapore | 64 | 2011/02/03 | $234,500 | 2120 | o.liang@datatables.net |
| Bruno | Nash | Software Engineer | London | 38 | 2011/05/03 | $163,500 | 6222 | b.nash@datatables.net |
| Sakura | Yamamoto | Support Engineer | Tokyo | 37 | 2009/08/19 | $139,575 | 9383 | s.yamamoto@datatables.net |
| Thor | Walton | Developer | New York | 61 | 2013/08/11 | $98,540 | 8327 | t.walton@datatables.net |
| Finn | Camacho | Support Engineer | San Francisco | 47 | 2009/07/07 | $87,500 | 2927 | f.camacho@datatables.net |
| Serge | Baldwin | Data Coordinator | Singapore | 64 | 2012/04/09 | $138,575 | 8352 | s.baldwin@datatables.net |
| Zenaida | Frank | Software Engineer | New York | 63 | 2010/01/04 | $125,250 | 7439 | z.frank@datatables.net |
| Zorita | Serrano | Software Engineer | San Francisco | 56 | 2012/06/01 | $115,000 | 4389 | z.serrano@datatables.net |
| Jennifer | Acosta | Junior Javascript Developer | Edinburgh | 43 | 2013/02/01 | $75,650 | 3431 | j.acosta@datatables.net |
| Cara | Stevens | Sales Assistant | New York | 46 | 2011/12/06 | $145,600 | 3990 | c.stevens@datatables.net |
| Hermione | Butler | Regional Director | London | 47 | 2011/03/21 | $356,250 | 1016 | h.butler@datatables.net |
| Lael | Greer | Systems Administrator | London | 21 | 2009/02/27 | $103,500 | 6733 | l.greer@datatables.net |
| Jonas | Alexander | Developer | San Francisco | 30 | 2010/07/14 | $86,500 | 8196 | j.alexander@datatables.net |
| Shad | Decker | Regional Director | Edinburgh | 51 | 2008/11/13 | $183,000 | 6373 | s.decker@datatables.net |
| Michael | Bruce | Javascript Developer | Singapore | 29 | 2011/06/27 | $183,000 | 5384 | m.bruce@datatables.net |
| Donna | Snider | Customer Support | New York | 27 | 2011/01/25 | $112,000 | 4226 | d.snider@datatables.net |

- Javascript
- HTML
- CSS
- Ajax
- Server-side script

The Javascript shown below is used to initialise the table shown in this
example:

`$(document).ready(function() {
var table = $('#example').DataTable( {
scrollY: "300px",
scrollX: true,
scrollCollapse: true,
paging: false
} );
new $.fn.dataTable.FixedColumns( table, {
heightMatch: 'none'
} );
} );`

In addition to the above code, the following Javascript library files are loaded for use in this
example:

- ../../../media/js/jquery.js
- ../../../media/js/jquery.dataTables.js
- ../js/dataTables.fixedColumns.js

The HTML shown below is the raw HTML table element, before it has been enhanced by
DataTables:

This example uses a little bit of additional CSS beyond what is loaded from the library
files (below), in order to correctly display the table. The additional CSS used is shown
below:

`/* Ensure that the demo table scrolls */
th, td { white-space: nowrap; }
div.dataTables_wrapper {
width: 800px;
margin: 0 auto;
}
tr { height: 50px; }`

The following CSS library files are loaded for use in this example to provide the styling of the
table:

- ../../../media/css/jquery.dataTables.css
- ../css/dataTables.fixedColumns.css

This table loads data by Ajax. The latest data that has been loaded is shown below. This data
will update automatically as any additional data is loaded.

The script used to perform the server-side processing for this table is shown below. Please note
that this is just an example script using PHP. Server-side processing scripts can be written in any
language, using the protocol described in the
DataTables documentation.

## Other examples

### Examples

- Left and right fixed columns
- Basic initialisation
- Multiple fixed columns
- Right column only
- Complex headers
- ColVis integration
- Server-side processing
- CSS row sizing
- Assigned column width
- Fluid column width
- Individual column filtering
- Bootstrap
- Index column

Please refer to the DataTables documentation for full
information about its API properties and methods.  
Additionally, there are a wide range of extras and
plug-ins which extend the capabilities of
DataTables.

DataTables designed and created by SpryMedia Ltd © 2007-2014  
DataTables is licensed under the MIT license.
